# A Case Report of Kikuchi Fujimoto Disease as an Antecedent Illness of Systemic Lupus Erythematosus in a Male

**DOI:** 10.7759/cureus.49693

**Published:** 2023-11-30

**Authors:** K. V. P. Munasinghe, M. A. V. M. U. Karunarathne, J. A. S. Sandamali, D. Munidasa

**Affiliations:** 1 General Medicine, Colombo South Teaching Hospital, Colombo, LKA; 2 Rheumatology, Rheumatology and Rehabilitation hospital, Ragama, Ragama, LKA; 3 Rheumatology, Rheumatology and Rehabilitation Hospital, Ragama, LKA

**Keywords:** antinuclear antibody, male, cervical lymphadenopathy, systemic lupus erythematosus, kikuchi fujimoto disease

## Abstract

Kikuchi Fujimoto disease (KFD) is a rare benign self-limiting condition described in young females characterized by lymphadenopathy and fever. It has been associated with several infective and autoimmune diseases, among which systemic lupus erythematosus (SLE) is relatively common. Kikuchi disease could occur either as a proceeding illness or as a coexisting illness with SLE. The presence of necrotizing lymphadenitis is appreciated in the histological specimen to confirm the diagnosis. Anti-nuclear antibody (ANA) positivity implicates a possible correlation with SLE or recurrence of the pre-existing disease. This clinical presentation needs to be evaluated thoroughly to prevent misdiagnosis and inappropriate treatment. Although Kikuchi disease generally warrants supportive treatment, steroids and immune therapy play a role in treating this persistent and recurrent disease. Long-term surveillance is mandatory for the early detection of sinister pathologies.

## Introduction

Kikuchi Fujimoto disease (KFD) is a benign condition that was initially reported in Japan, hence its name [1]. It is a rare disease entity, also termed Kikuchi disease or Kikuchi histiocytic necrotizing lymphadenitis, which is characterized by fever and cervical lymphadenopathy in a young female [2]. The median age of presentation is 30 years. Despite its enigmatic pathogenesis, histological evidence is in favor of a T-cell-mediated immune response to an infectious agent. According to recent literature, the prevalence of Kikuchi disease in males is nearly equal to that in females [1,3]. Other than systemic lupus erythematosus (SLE), Kikuchi disease is also associated with several autoimmune and infectious diseases. The published reviews estimate that in 30% of cases, KFD proceeds with SLE, whereas in 23% of cases, it follows SLE [4].

SLE is an autoimmune disease with multi-system involvement commonly described in females of childbearing age. Estrogen hormonal effects, environmental factors, and genetic factors concerning the X chromosome have been attributed to the female preponderance of SLE, hence the female-to-male ratio ranging from 7:1 to 15:1 [5]. The disease onset in men is generally delayed compared to women, and 40 indicates the mean age of onset for men. We hereby report a case of a previously healthy adolescent male presenting with biopsy-proven Kikuchi disease, which acts as the antecedent illness of systemic lupus erythematosus.

## Case presentation

A 15-year-old previously healthy boy presented with a high-grade intermittent fever responsive to antipyretics and a chronic dry cough over a period of two months. Other than the nonspecific body aches that accompanied the fever, he was systemically well. One week later, he was noted to have multiple non-tender, rubbery lumps in the cervical region bilaterally in favor of cervical lymphadenopathy. He did not complain of constitutional symptoms, including loss of appetite and loss of weight. No history of easy fatigability; prone to recurrent infections or skin rashes. Neither a history of exposure to tuberculosis nor a family history of malignancies and autoimmune disorders could be appreciated. He denied a recent travel history, sexual promiscuity, and the use of recreational drugs, alcohol, or tobacco. There was no exposure to domestic pets, specifically cats. Upon seeking medical attention during the initial presentation, he was subjected to a cervical lymph node excision biopsy, which was histologically more in favor of Kikuchi lymphadenitis.

One month later, he presented with persistent fever spikes and oral ulcers. There were no joint symptoms, discoloration of digits on cold exposure, change in skin integrity, muscle weakness, reduced lacrimation, or salivation.

**Figure 1:**
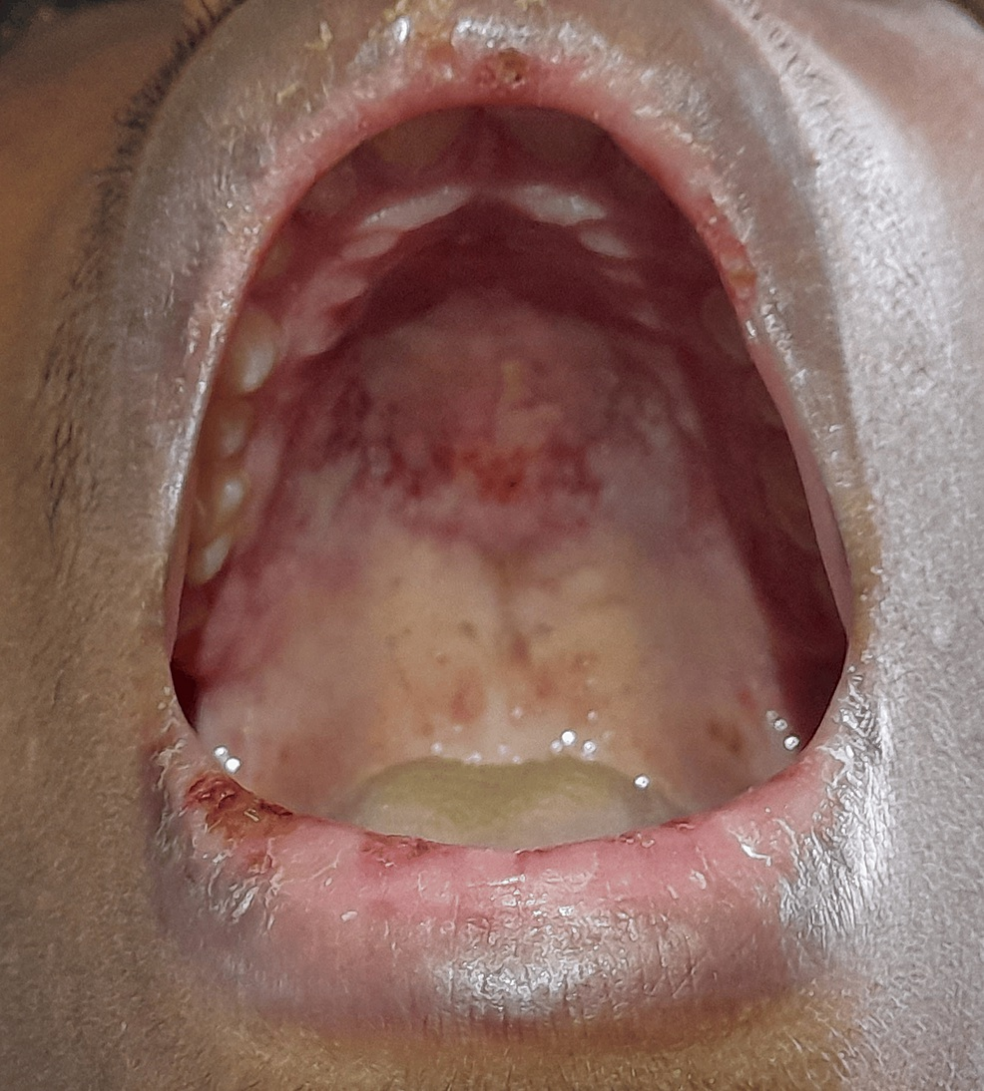
Palatal petechiae with ulcers in the oral mucosa

**Figure 2:**
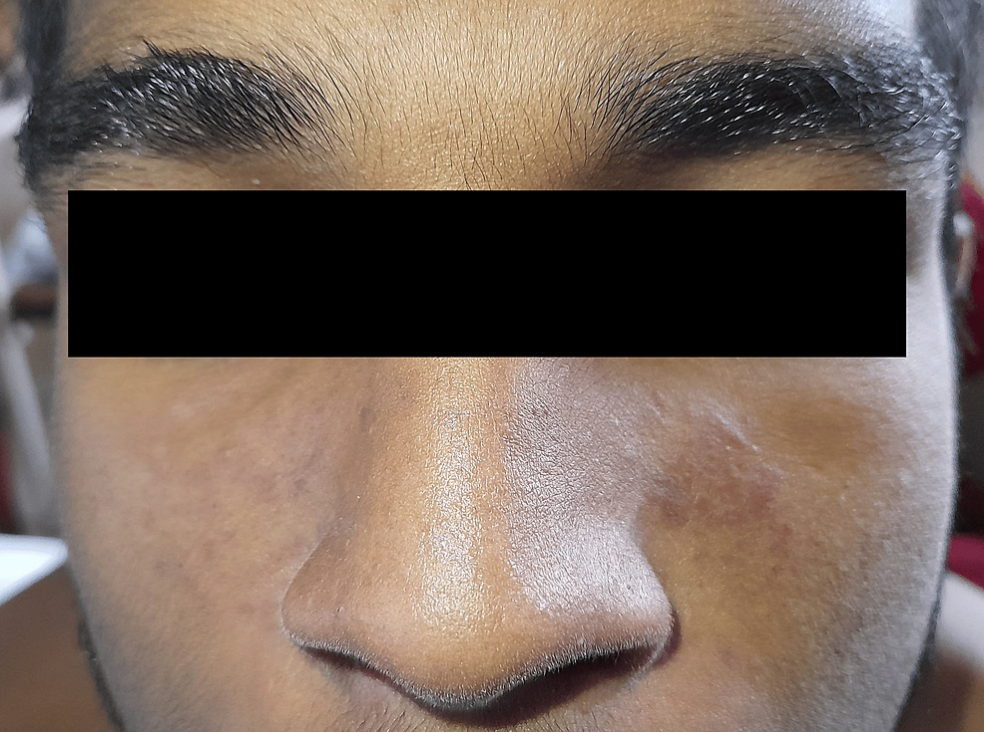
Malar rash in systemic lupus erythematosus sparing the nasolabial folds

On examination, he was not pale or icteric. There was bilateral cervical lymphadenopathy in the anterior and posterior triangles and bilateral axillary lymphadenopathy. Multiple oral ulcers and palatal petechiae (Figure 1) were noted. On thorough inspection, a rash sparing the nasolabial folds (Figure 2), which was difficult to discern with the original complexion of the boy, and areas of patchy alopecia over the scalp were noted. The rest of the examination, including musculoskeletal, cardiovascular, respiratory, abdominal, neurological, and ophthalmological examinations, was unremarkable.

**Figure 3:**
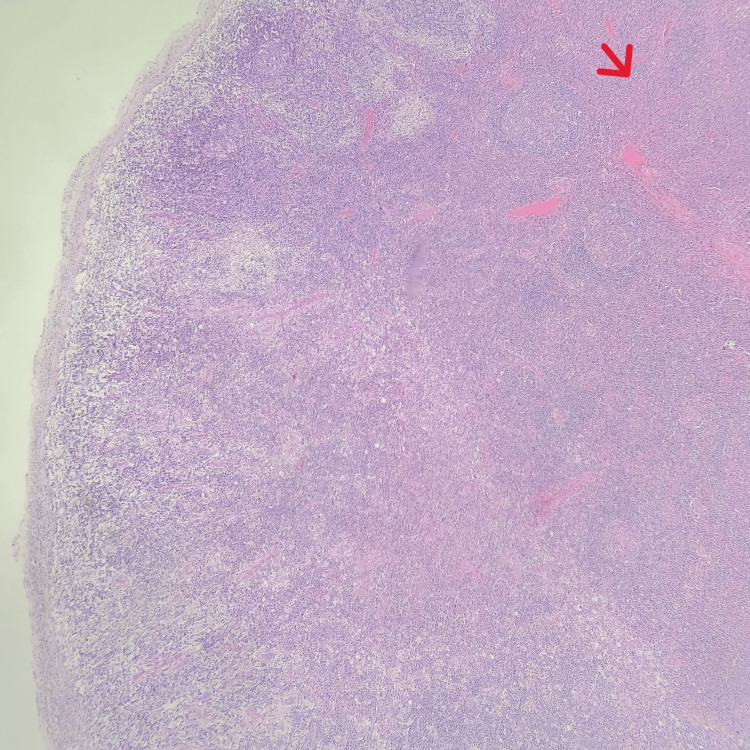
Hematoxylin and eosin stained lymph node showing distorted architecture by confluent areas of necrosis (red arrow)

**Figure 4:**
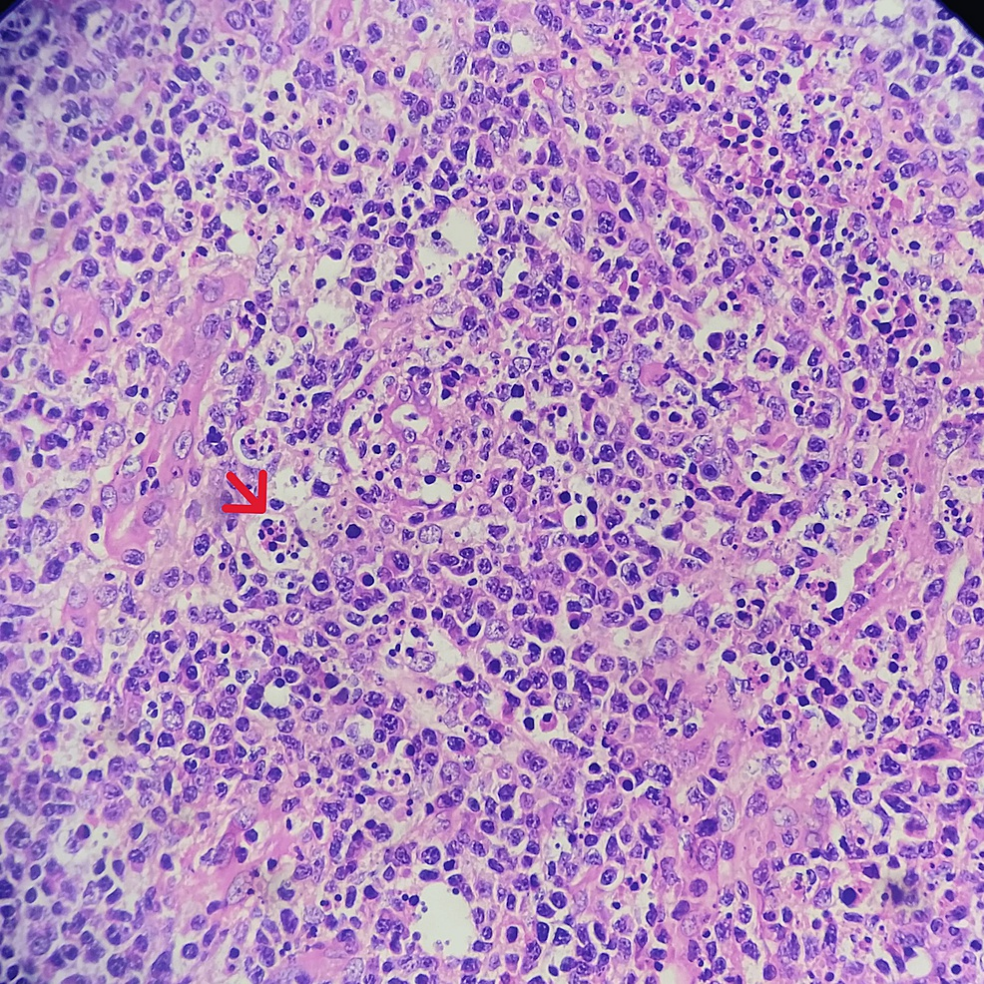
Pancortex expansion of the lymph node showing increased histiocytes containing karyorrhectic nuclear debris (red arrow) and plasmacytoid dendritic cells

His investigations (Table 1) revealed bicytopenia with a low white blood cell (WBC) and platelet count. The erythrocyte sedimentation rate (ESR) was 55 mm/hour. There was no biochemical evidence of an acute infection. The blood picture showed infection or inflammation with no abnormal cells. The tuberculosis screening was negative. Lactate dehydrogenase (LDH) was 341 IU/L. Epstein-Barr virus (EBV), rickettsial, and retroviral screening were negative. Chest X-rays (CXR) revealed no mediastinal widening and normal lung parenchyma. A 2D echocardiogram revealed good biventricular function with no vegetation and an ejection fraction of 60%. An ultrasound scan (USS) of the neck revealed multiple enlarged reactive lymph nodes in the cervical region bilaterally at levels II, III, and IV. A cervical lymph node biopsy revealed necrotizing lymphadenitis more in favor of Kikuchi lymphadenitis (Figures 3-4). An ultrasound scan of the abdomen and contrast-enhanced computer tomography of the chest, abdomen, and pelvis (CECT CAP) were unremarkable.

**Table 1:** Laboratory parameters WBC: white blood cells, CPR: c-reactive protein, ESR: erythrocyte sedimentation rate, LDH: lactate dehydrogenase, AST: aspartate aminotransferase, ALT: alanine aminotransferase, FBS: fasting blood sugar, AFB: acid fast bacillus, UFR: urine full report, ACR: albumin creatinine ratio, EBV: Epstein-Barr virus, ANA: anti-nuclear antibody, C: complement.

Investigation	Values	Reference range
WBC	2.69 x 10^9^/L	4–10 x 10^9^/L
Neutrophils	1.1 × 10^9^/L	2–7 x 10^9^/L
Lymphocytes	1.29 × 10^9^/L	1–3 x 10^9^/L
Hemoglobin	11.9 g/dL	11–16 g/dL
Platelets	111 x 10^9^/L	150–450 x 10^9^/L
Serum sodium	140 mmol/L	Na – 135–145 mmol/L
Serum potassium	4.6 mmol/L	K – 3.5–5.5 mmol/L
CRP	1.4 mg/dL	<6
ESR	55 mm/hour	<20 mm/hour
LDH	341 IU/L	105–333 IU/L
AST	30 U/L	8–33 U/L
ALT	24 U/L	4–36 U/L
Serum albumin	4.6 g/dL	3.4–5.4 g/dL
Total bilirubin	1.2 mg/dL	0.1–1.2 mg/dL
Renal function tests	S. creatinine – 1 mg/dL	0.7–1.3 mg/dL
FBS	88 mg/dL	74–100 mg/dL
Mantoux test	Negative	
Sputum AFB	Negative	
UFR	Protein nil, no pus cells, no red cells	
Urine ACR	25.2 mg/g	<30 mg/g
Retroviral studies	Negative	
EBV	Negative	
Rickettsial screening	Negative	
ANA	1:1280, nuclear homogenous pattern	1:160 positive
C3	75 mg/dL	90–180 mg/dL
C4	8 mg/dL	10–40 mg/dL

At the end of the exhaustive evaluation, Kikuchi's disease was confirmed after safely excluding infectious and neoplastic causes, and concerning the clinical picture during the second presentation, a possible inflammatory/connective tissue pathology was suspected. The anti-nuclear antibody (ANA) test yielded a highly positive titer of a 1:1280 nuclear homogenous pattern. With the economic constraints, the patient could not afford other specific autoantibody testing for SLE, such as anti-double-stranded deoxyribonucleic acid antibodies (ds DNA) or anti-Smith antibodies (anti-SM). However, the patient met the diagnostic criteria for systemic lupus erythematosus by scoring 15 points according to the 2019 European Alliance of Associations for Rheumatology (EULAR)/American College of Rheumatology (ACR) classification criteria. Low C3 and C4 levels supported the diagnosis of a SLE flare.

He was pulsed with methylprednisolone 1 mg/kg for three days, followed by oral prednisolone 0.5 mg/kg/day and hydroxychloroquine 5 mg/kg/day, following which his fever completely subsided and was clinically improved by the time he was discharged. A routine rheumatology clinic follow-up was arranged.

## Discussion

Kikuchi disease is a benign, self-limiting illness predominantly described in young females. Lymphadenopathy (100%) is the most commonly reported clinical manifestation, primarily involving the cervical nodes, followed by fever (35%). Other signs and symptoms such as rash, arthritis, fatiguability, and hepatosplenomegaly have also been reported, but infrequently [6]. The diagnosis of Kikuchi disease is made by a lymph node biopsy, preferably an excisional biopsy, and histological analysis. Ultrasound-guided fine needle aspiration is an alternative diagnostic method [7]. A microscopic examination shows paracortical foci with necrosis and a histiocytic cellular infiltrate.

SLE, a connective tissue disorder with multi-systemic involvement, has been described simultaneously with Kikuchi's disease. Other than SLE, adult-onset Still’s disease, SARS-CoV-2 infection, and Sjogren disease are a few disease entities reported to coexist with Kikuchi disease in the literature [8-10]. Serology for Epstein-Barr virus, cytomegalovirus, HIV, toxoplasmosis, *Yersinia enterocolitica*, and cat scratch disease is often done to exclude infection-related fever and lymphadenopathy [4]. The difference between Kikuchi and SLE is that Kikuchi presents with prominently cervical lymphadenopathy with rarely positive ANA and a self-limiting nature, whereas in SLE, ANA positivity predominates and needs specific treatment.

During the patient's initial presentation, we arrived at a few differential diagnoses. Tuberculosis was a priority, which was excluded with the negative results on the Mantoux test, sputum studies, and lymph node biopsy for granulomatous inflammation. The possibility of lymphoma was also excluded with the lymph node biopsy and extensive imaging of the entire body. Other common viral etiologies were excluded following serology for EBV, rickettsia, and retroviral studies. Chest X-rays excluded hilar lymphadenopathy and hence sarcoidosis, and the diagnosis of Kikuchi was reinforced by a pathognomonic lymph node biopsy.

Since Kikuchi disease is self-limiting, no effective treatment is needed, and resolution generally occurs within one to four months [11]. For patients with persistent symptoms, the described treatment options are glucocorticoids or high-dose glucocorticoids with intravenous immune globulin (IV IG) [12]. The literature describes cases of recurrent Kikuchi disease treated with hydroxychloroquine monotherapy or combined with glucocorticoids or an interleukin-1 inhibitor, anakinra, for steroid non-respondents [13,14].

## Conclusions

Kikuchi disease is a benign disease that is self-limiting in the majority, despite the recurrences reported. A positive fluorescent ANA is associated with an increased recurrence rate. Not that KFD always presents as the antecedent illness of SLE; it can coexist with SLE, giving rise to a diagnostic dilemma. It is important to be aware of this presentation to prevent misdiagnosis and inappropriate treatment. An extensive evaluation is recommended, excluding possible infective, autoimmune, and malignant causes. Although, in most cases, supportive therapy is needed, steroids, IV IG, hydroxychloroquine, and anakinra play an important role in persistent and recurrent disease. Long-term surveillance is important for the early detection of other sinister pathologies.
